# Upadacitinib Reduces Rates of Hospitalization in Ulcerative Colitis: A Large-Scale Nationwide Cohort Study

**DOI:** 10.3390/jcm15145507

**Published:** 2026-07-14

**Authors:** Chiaki Maeyashiki, Nobuharu Tamaki, Yuki Tanaka, Yutaka Yasui, Kaoru Tsuchiya, Hiroyuki Nakanishi, Masayuki Kurosaki

**Affiliations:** Department of Gastroenterology and Hepatology, Musashino Red Cross Hospital, Tokyo 180-8610, Japan; verde.ch1ak1@gmail.com (C.M.); nobuharu.tamaki@gmail.com (N.T.); lavi.scarlet@gmail.com (Y.T.); yutakayas@hotmail.com (Y.Y.); tsuchiyakaoru3@gmail.com (K.T.); hnnakanishi@gmail.com (H.N.)

**Keywords:** ulcerative colitis, upadacitinib, hospitalization

## Abstract

**Background/Objectives:** Upadacitinib is recommended for moderate-to-severe ulcerative colitis (UC); however, data regarding its effectiveness and long-term outcomes in large patient populations remain limited. In this nationwide, hospital-based cohort study, we investigated the effectiveness and long-term outcomes of upadacitinib in patients with UC. **Methods:** This study included 2694 patients with UC who received upadacitinib. The primary outcome was the comparison of hospitalization rates during the 1-year periods before and after the initiation of upadacitinib. Additionally, the cumulative hospitalization rate up to 2 years post-initiation was evaluated. **Results:** Of 2694 patients who were initiated on upadacitinib, the median age was 45 (31–57) years, and 60.5% were male. In the year preceding upadacitinib initiation, 29.3% of the patients were hospitalized due to UC. In the 1 year following upadacitinib initiation, the hospitalization rate significantly decreased to 9.6% (*p* < 0.001). Furthermore, the cumulative hospitalization rate up to 2 years was 12.6%. Among patients with a prior history of hospitalization, the hospitalization rate in the 1 year after upadacitinib initiation decreased from 100% to 16.8%. A significant improvement in the hospitalization rate was observed in both advanced therapy-naïve and -exposed patients. After adjusting for age, sex, steroid use, and history of advanced therapy, the hazard ratio (95% confidence interval) for hospitalization associated with upadacitinib initiation was 0.39 (0.33–0.46, *p* < 0.001). **Conclusions:** The initiation of upadacitinib was associated with significantly lower hospitalization rates, and this association was maintained for up to two years. These findings support upadacitinib as an effective and viable treatment option for patients with moderate-to-severe UC.

## 1. Introduction

Ulcerative colitis (UC) is a chronic, relapsing, and remitting inflammatory bowel disease that affects the rectum and colon [1]. Because the disease frequently affects younger individuals and can lead to repeated hospitalizations or the need for surgery, the resulting decline in quality of life (QOL) is a major concern [2,3]. Furthermore, the global prevalence of UC has been increasing in recent years, with a particularly notable rise in Asia [4,5]. Consequently, optimizing treatment strategies for this disease has become a critical challenge.

In recent years, advances in therapeutics for moderate-to-severe UC have made various advanced therapies, such as biologics and small-molecule compounds, available in clinical practice [6,7]. These pharmacological advancements have improved treatment outcomes in refractory cases, leading to decreases in both surgical and hospitalization rates [8,9,10].

Upadacitinib, a Janus kinase (JAK) inhibitor, demonstrated significant efficacy in inducing clinical remission compared to placebo in Phase 3 clinical trials, leading to its regulatory approval [11]. The effectiveness of upadacitinib in real-world clinical settings has also been demonstrated across multiple studies. Currently, various clinical guidelines recommend upadacitinib for moderate-to-severe UC patients [6,12]. Moreover, some reports suggest it yields higher therapeutic efficacy than other advanced therapies, positioning it as a potentially highly effective treatment option [13,14,15].

However, because the time elapsed since its approval is relatively short, real-world data regarding its effectiveness and long-term outcomes in large patient populations remain limited, highlighting the need to gather further evidence. Although several real-world observational studies have reported the efficacy of upadacitinib, the majority of these reports are limited to small cohorts, typically ranging from a few dozen to a few hundred patients [16,17,18,19]. Furthermore, whereas many studies have evaluated its effectiveness over a one-year period, reports examining long-term effectiveness remain scarce. To fill this knowledge gap, the present study aimed to elucidate the real-world treatment outcomes of upadacitinib in a large-scale UC cohort using a nationwide medical claims database in Japan, representing one of the largest evaluations to date.

## 2. Methods

### 2.1. Data Sources

This study is a nationwide, hospital-based cohort analysis utilizing the Medical Data Vision (MDV) database, managed by MDV Co., Ltd., Tokyo, Japan [20]. The database compiles monthly claims from acute care/tertiary hospitals as well as pharmacies. In March 2026, the MDV database included data from 616 acute care/tertiary hospitals in Japan, which accounts for approximately 35% of all Japanese acute care/tertiary hospitals, reporting the status and treatment of approximately 57 million patients. The available information encompasses medical and pharmacy claims, including diagnoses, medications, clinical procedures and treatments, and Japan’s unique diagnosis procedure combination system for both inpatient and outpatient services. By covering such a substantial proportion of all Japanese acute care and tertiary hospitals, this extensive dataset allows for the evaluation of large-scale real-world clinical practice. Unlike previous single-center or small multi-center real-world studies, the use of this nationwide database enabled us to gather data from over 2000 patients, thereby minimizing selection bias and reflecting a truly representative, nationwide clinical landscape.

In the database, diagnoses are classified according to the International Classification of Diseases, 10th revision (ICD-10). Clinical practice data, which cover examinations, treatments, and surgeries, are coded using Japan’s specific procedural coding system.

### 2.2. Study Protocol

**Figure 1:**
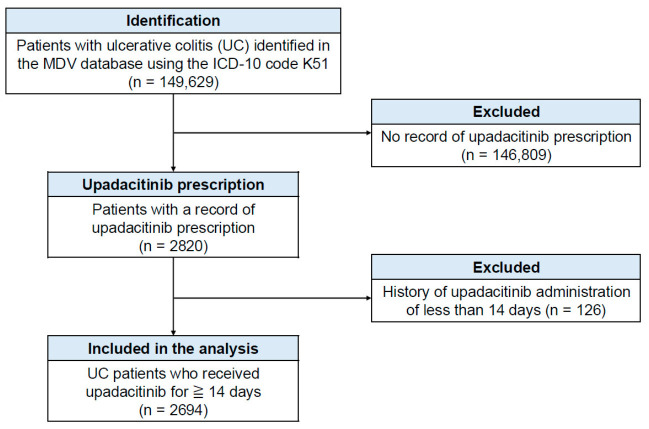
Flowchart of patient selection.

A total of 149,629 patients with UC were identified in the MDV database up to December 2025 using the ICD-10 code K51. Among them, 2820 patients had a record of upadacitinib prescription. Because clinical trials have reported that it takes 2 weeks for the therapeutic efficacy of upadacitinib to manifest [11,21,22], patients with a history of upadacitinib administration of less than 14 days were excluded. Consequently, 2694 UC patients who received upadacitinib were included in the analysis (Figure 1).

This study was conducted in accordance with the ethical guidelines outlined in the Declaration of Helsinki. Because the MDV database contains only anonymized data, the requirements for informed consent and institutional review board approval were waived.

### 2.3. Primary Endpoint

The primary endpoint of this study was hospitalization due to UC. Hospitalization is a clinically meaningful outcome that reflects disease severity and inadequate treatment response, while directly affecting patients’ QOL. In addition, hospitalization events can be reliably ascertained from claims databases. Therefore, hospitalization was chosen as the primary outcome of this study.

Hospitalization rates during the 1 year prior to and 1 year after the initiation of upadacitinib were compared. As a secondary outcome, the hospitalization rate up to 2 years after initiation was also evaluated.

### 2.4. Definition of Events

All hospitalization records are documented in the database along with primary diagnoses. Therefore, hospitalization due to UC was defined as an admission with a primary diagnosis of UC (ICD-10 code K51). Regarding surgeries, using Japan’s specific procedural coding system, patients with a code for colectomy (K719) and a primary diagnosis of UC were defined as having undergone a bowel resection.

### 2.5. Medication Use

Upadacitinib use was identified using the 9-digit Receipt Computer Processing System Codes (7.5 mg tablet: 622793901; 15 mg tablet: 622793902; 30 mg tablet: 622793903; and 45 mg tablet: 622793904). Exposure to biologic agents and small-molecule therapies was identified using the corresponding drug codes. Patients who received each medication for ≥14 days were classified as having received the respective therapy.

Corticosteroid use was identified using the European Pharmaceutical Market Research Association (EphMRA) Anatomical Therapeutic Chemical (ATC) Classification System, specifically H02A1 (injectable corticosteroids) and H02A2 (oral corticosteroids). Patients who received corticosteroids for ≥28 days were classified as corticosteroid users.

### 2.6. Statistical Analysis

Continuous variables were expressed as medians with interquartile ranges (IQRs). Categorical variables were presented as numbers and percentages. Subgroup analyses were specifically stratified by prior advanced therapy status to independently assess clinical outcomes in both advanced therapy-naïve and advanced therapy-exposed populations. Hospitalization rates after upadacitinib initiation were calculated using the Kaplan–Meier method. Similarly, the colectomy rate was evaluated using the Kaplan–Meier method. Changes in the hospitalization rate before and after upadacitinib initiation were compared using a marginal Cox model. The hazard ratio for the risk of hospitalization due to upadacitinib initiation was calculated using a marginal Cox model, adjusted for age, sex, steroid use, and history of advanced therapy. A major limitation of the claims database is the lack of detailed clinical data, such as endoscopic findings and disease severity scores. Consequently, direct comparisons between different advanced therapies could introduce severe unmeasured confounding by indication. To mitigate this bias, we employed a self-controlled design, which allows for the implicit adjustment of patient-specific, time-invariant unmeasured confounders. A *p*-value < 0.05 was considered statistically significant. All statistical analyses were performed using EZR (Saitama Medical Center, Jichi Medical University, Shimotsuke, Japan), a graphical user interface for R version 3.2.2 (The R Foundation for Statistical Computing, Vienna, Austria).

Adherence was assessed using the Proportion of Days Covered (PDC) method. It was calculated by dividing the total number of dispensed days during the upadacitinib prescription period by the observation period, and a PDC of ≥80% was defined as high adherence [23].

### 2.7. Use of AI Tools

Google Gemini 3.1 was used for English language editing. AI tools were not used for data collection, statistical analysis, or manuscript preparation. The authors have reviewed and edited the output and take full responsibility for the content of this publication.

## 3. Results

### 3.1. Patient Characteristics

This study included 2694 patients who were initiated on upadacitinib for UC (Table 1). The median age (IQR) was 45 (31–57) years, and 60.5% were male. Among the included patients, 60.8% had received advanced therapy prior to upadacitinib initiation. The most common advanced therapies administered immediately prior to upadacitinib initiation were infliximab (11.0%), vedolizumab (10.1%), filgotinib (9.1%), and ustekinumab (9.0%).

### 3.2. Changes in Hospitalization Rates Following Upadacitinib Initiation

**Figure 2:**
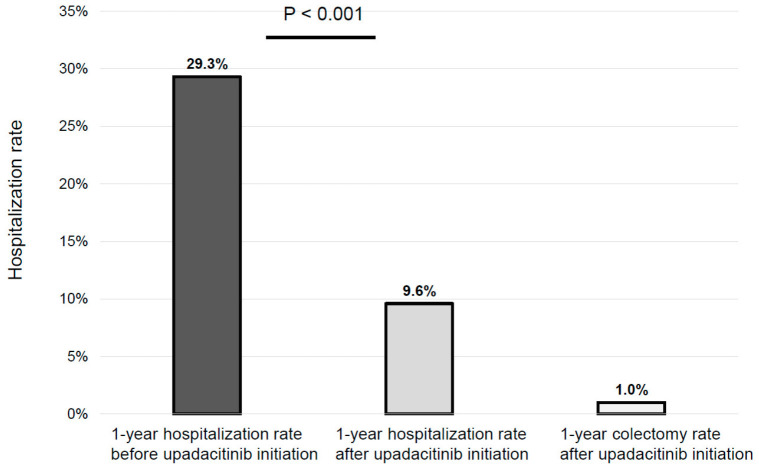
Changes in hospitalization and colectomy rates following upadacitinib initiation.

During the 1 year prior to upadacitinib initiation, 788 patients were hospitalized because of UC, whereas 235 patients were hospitalized during the 1 year following upadacitinib initiation. The 1-year cumulative hospitalization rate significantly decreased from 29.3% before upadacitinib initiation to 9.6% after initiation (*p* < 0.001, Figure 2). Additionally, 1.0% of the patients required a colectomy within 1 year after the initiation of upadacitinib.

### 3.3. Subgroup Analysis Based on Prior Advanced Therapy or Prior Hospitalization

**Figure 3:**
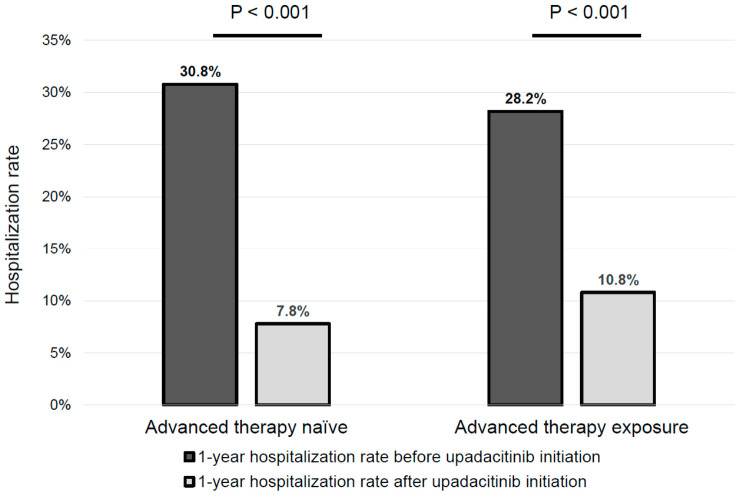
Changes in hospitalization rates following upadacitinib initiation, stratified by prior advanced therapy status.

A subgroup analysis was conducted based on prior advanced therapy status. In advanced therapy-naïve patients (39.2% of the cohort), the hospitalization rate in the 1 year prior to upadacitinib initiation was 30.8%. Following upadacitinib initiation, the hospitalization rate in the subsequent 1 year significantly decreased to 7.8% (*p* < 0.001, Figure 3). Similarly, when examining the hospitalization rate in advanced therapy-exposed patients (60.8% of the cohort), a significant improvement was observed, dropping from 28.2% in the 1 year before initiation to 10.8% in the 1 year after initiation (*p* < 0.001).

Furthermore, a subgroup analysis was performed, stratified by a history of hospitalization prior to upadacitinib initiation. Among patients with a prior history of hospitalization, the hospitalization rate in the 1 year after upadacitinib initiation decreased from 100% to 16.8%. Additionally, among patients without a history of hospitalization prior to upadacitinib initiation, the 1-year hospitalization rate was 6.7%.

### 3.4. Hospitalization Rates up to 2 Years After Upadacitinib Initiation

**Figure 4:**
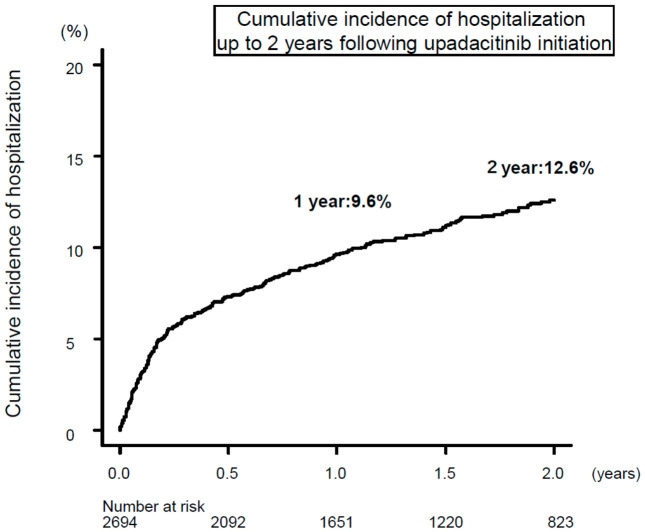
Cumulative incidence of hospitalization up to 2 years following upadacitinib initiation.

The hospitalization rate up to 2 years after upadacitinib initiation was evaluated. The hospitalization rate at 1 year after initiation was 9.6% (Figure 4). When the observation period was extended to 2 years, the cumulative hospitalization rate was 12.6%.

### 3.5. Changes in the Risk of Hospitalization by Upadacitinib Initiation

**Figure 5:**
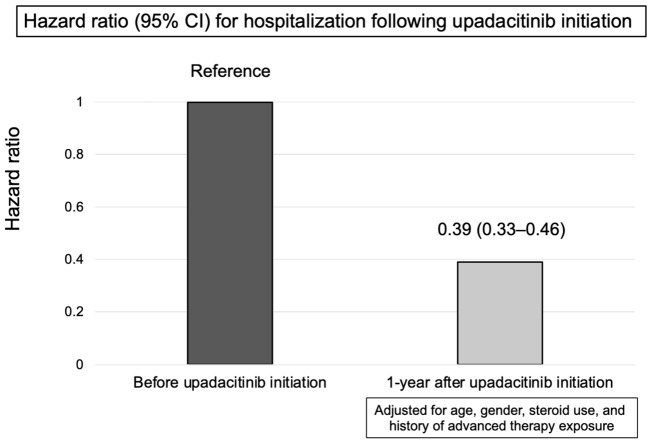
Hazard ratios for hospitalization following upadacitinib initiation.

Using the pre-initiation period as a reference, changes in the risk of hospitalization following upadacitinib initiation were evaluated. After adjusting for age, sex, steroid use, and history of advanced therapy, the hazard ratio (95% confidence interval) for hospitalization associated with upadacitinib initiation was 0.39 (0.33–0.46, *p* < 0.001, Figure 5), indicating a significant reduction in the risk of hospitalization.

### 3.6. Medication Continuation Rate

The medication continuation rate for upadacitinib was 73.8% at 1 year and 61.2% at 2 years. Regarding the PDC, all patients who continued therapy maintained a PDC of ≥80%, confirming consistent adherence throughout the observation period.

## 4. Discussion

### 4.1. Main Findings

This study elucidated the treatment outcomes of upadacitinib in over 2600 patients with UC. The introduction of upadacitinib was associated with lower hospitalization rates, and its therapeutic effects were shown to be maintained for up to two years. Furthermore, it was effective not only in advanced therapy-naïve patients but also in those with prior exposure to advanced therapies. Upadacitinib is currently recommended by various guidelines as a treatment for moderate-to-severe UC [6,12]. Our findings further support these recommendations, confirming that upadacitinib is a highly viable and practical treatment option for this patient population.

### 4.2. Comparison with Previous Literature

Upadacitinib has been shown to be effective not only in advanced therapy-naïve cases but also in high-risk patient populations, such as those with biologic-refractory or steroid-resistant disease [6,24]. Its efficacy has been demonstrated not only in clinical trials but also across various cohort studies [11,16,18]. However, compared to earlier-approved agents like anti-TNFα antibodies (e.g., infliximab, adalimumab), large-scale studies evaluating the real-world efficacy of the recently approved upadacitinib remain limited. Previous real-world observational studies assessing upadacitinib have largely been constrained by sample sizes ranging from several dozen to a few hundred patients [11,16,18]. The unprecedented scale of our cohort—exceeding 2600 patients—marks it as one of the largest real-world evaluations of upadacitinib to date. This massive scale provides a significant advantage, affording substantial statistical power to validate the outcomes observed in smaller cohorts while mitigating institutional biases.

Clinical trials for upadacitinib primarily evaluated clinical remission at 52 weeks as the primary outcome [11,25]. Similarly, the majority of hospital-based cohort studies have mainly reported one-year treatment outcomes [17,18,19]. While three-year follow-up data from clinical trials have demonstrated long-term efficacy [26], reports detailing long-term, real-world outcomes remain exceedingly limited due to the short time since the drug’s approval. Therefore, this study provides important real-world evidence regarding the two-year effectiveness of upadacitinib.

Furthermore, clinical trials and studies on advanced therapies often utilize clinical or endoscopic remission as primary outcomes [27]. In contrast, few studies have focused on hospitalization as a primary endpoint [17]. Because hospitalization significantly impairs patient QOL and represents a major adverse outcome [28], verifying whether advanced therapies actually reduce real-world hospital admissions is crucial. The clear demonstration that upadacitinib lowers hospitalization rates further substantiates its overall clinical value. From a healthcare system perspective, hospital admissions also account for a considerable proportion of the economic burden associated with UC. Therefore, the marked decrease in hospitalization rates observed in our cohort suggests that the benefits of upadacitinib extend beyond symptom control and may translate into meaningful improvements in patient well-being and healthcare resource utilization.

Upadacitinib has demonstrated efficacy for both induction and maintenance therapy in Crohn’s disease in phase 3 clinical trials [29]. However, because its approval for Crohn’s disease followed that for ulcerative colitis, real-world evidence regarding clinically relevant outcomes, such as hospitalization and surgery, remains scarce. Likewise, although upadacitinib had already been approved for several immune-mediated inflammatory diseases, including rheumatoid arthritis [30], psoriatic arthritis [31], axial spondyloarthritis [32], and atopic dermatitis [33], before its approval for ulcerative colitis, evidence supporting its long-term effectiveness in ulcerative colitis—particularly with respect to clinically meaningful outcomes—has been limited.

Therefore, the demonstration of the two-year maintenance effectiveness of upadacitinib in ulcerative colitis represents an important value of the present study.

Upadacitinib has not yet been approved for the treatment of pediatric ulcerative colitis either in Japan or internationally. Accordingly, evidence regarding its efficacy and safety in pediatric patients remains scarce. However, although a multicenter study has suggested favorable outcomes with upadacitinib in pediatric ulcerative colitis [34], robust clinical evidence is lacking. Future studies should investigate the efficacy of upadacitinib in achieving steroid-free remission, as well as its long-term safety, in the pediatric population.

### 4.3. Strengths and Limitations

A major strength of this study is the investigation of a large-scale cohort of over 2600 UC patients treated with upadacitinib, utilizing a nationwide Japanese medical claims database. As highlighted earlier, distinguishing this study from prior research involving tens to hundreds of patients, the sheer size of the cohort substantially boosts the reliability of the observed real-world effectiveness. Because all hospitalization and prescription histories are comprehensively recorded, the ability to accurately trace clinical courses over time is a distinct advantage of using claims data.

However, this study also has several limitations. As an observational study using claims data, it cannot entirely eliminate selection bias. Information regarding disease duration, disease extent, endoscopic severity, laboratory findings, patient-reported outcomes, and other potentially important clinical characteristics was unavailable in the claims database. Therefore, residual confounding could not be completely excluded despite statistical adjustment.

In addition, this study employed a before–after observational design without a concurrent control group. Therefore, causal relationships between upadacitinib initiation and reduced hospitalization rates cannot be established. Although hospitalization rates declined after treatment initiation, the observed association may have been influenced by regression to the mean, natural fluctuations in disease activity, optimization of concomitant therapies, or other unmeasured factors. Accordingly, these findings should be interpreted as an association rather than definitive evidence of a causal treatment effect.

Furthermore, hospitalization practices may vary across healthcare systems, depending on differences in healthcare accessibility, admission thresholds, and institutional policies. Because this study was conducted using a nationwide Japanese claims database within the context of Japan’s universal healthcare system, the generalizability of these findings to other healthcare settings should be interpreted with caution.

Although JAK inhibitors have been associated with safety concerns, including herpes zoster and venous thromboembolism, safety outcomes were not evaluated in the present study. Further studies are warranted to assess the long-term safety profile of upadacitinib in real-world clinical practice.

### 4.4. Future Implications

This study demonstrated that upadacitinib reduces hospitalizations in patients with UC and that its therapeutic effects are maintained over a two-year period. Currently, various guidelines recommend multiple advanced therapies for moderate-to-severe UC, applicable to both advanced therapy-naïve and advanced therapy-failure cases [6]. The British Society of Gastroenterology guidelines position upadacitinib as a highly effective treatment [35]. However, clear criteria for selecting a specific agent among the diverse array of available advanced therapies have yet to be fully established. The findings of this study further reinforce the robust therapeutic efficacy of upadacitinib. While our findings robustly support its use, future research must prioritize head-to-head real-world comparative studies among available advanced therapies to establish optimal treatment sequencing.

In conclusion, in this large nationwide real-world cohort, upadacitinib initiation was associated with lower hospitalization rates that were maintained for up to two years. These findings support upadacitinib as an effective treatment option for patients with moderate-to-severe UC, regardless of prior advanced therapy exposure.

## Figures and Tables

**Table 1:** Patient characteristics.

			Overall
			*n* = 2694
Age, years			45 (31–57)
Gender	Male		1629 (60.5%)
	Female		1065 (39.5%)
Corticosteroid use	Before initiation of UPA		1077 (40.0%)
	After initiation of UPA		733 (27.2%)
Advanced therapies immediately prior to upadacitinib	None		1055 (39.2%)
	anti-TNF	infliximab	295 (11.0%)
		adalimumab	237 (8.8%)
		golimumab	106 (3.9%)
	anti-IL-12/23	ustekinumab	243 (9.0%)
	anti-IL-23	risankizumab	24 (0.9%)
		guselkumab	3 (0.1%)
		mirikizumab	38 (1.4%)
	anti-integrin	vedolizumab	271 (10.1%)
	JAK inhibitor	tofacitinib	173 (6.4%)
		filgotinib	246 (9.1%)
	S1P receptor modulator	ozanimod	3 (0.1%)

Continuous variables were expressed as medians with interquartile ranges. Categorical variables were presented as numbers and percentages. Corticosteroid use ≥28 days was defined as corticosteroid therapy for 28 days or longer during the one-year period before or after upadacitinib initiation.

## Data Availability

The data that support the findings of this study are available from Medical Data Vision but restrictions apply to the availability of these data, which were used under license for the current study and therefore are not publicly available.

## References

[1] Kobayashi T., Siegmund B., Le Berre C., Wei S.C., Ferrante M., Shen B., Bernstein C.N., Danese S., Peyrin-Biroulet L., Hibi T. (2020). Ulcerative colitis. Nat. Rev. Dis. Primers.

[2] Turner D., Ricciuto A., Lewis A., D’Amico F., Dhaliwal J., Griffiths A.M., Bettenworth D., Sandborn W.J., Sands B.E., Reinisch W. (2021). STRIDE-II: An Update on the Selecting Therapeutic Targets in Inflammatory Bowel Disease (STRIDE) Initiative of the International Organization for the Study of IBD (IOIBD): Determining Therapeutic Goals for Treat-to-Target strategies in IBD. Gastroenterology.

[3] Fumery M., Singh S., Dulai P.S., Gower-Rousseau C., Peyrin-Biroulet L., Sandborn W.J. (2018). Natural History of Adult Ulcerative Colitis in Population-based Cohorts: A Systematic Review. Clin. Gastroenterol. Hepatol..

[4] Ng S.C., Shi H.Y., Hamidi N., Underwood F.E., Tang W., Benchimol E.I., Panaccione R., Ghosh S., Wu J.C.Y., Chan F.K.L. (2017). Worldwide incidence and prevalence of inflammatory bowel disease in the 21st century: A systematic review of population-based studies. Lancet.

[5] Wei S.C., Sollano J., Hui Y.T., Yu W., Santos Estrella P.V., Llamado L.J.Q., Koram N. (2021). Epidemiology, burden of disease, and unmet needs in the treatment of ulcerative colitis in Asia. Expert. Rev. Gastroenterol. Hepatol..

[6] Singh S., Loftus E.V., Limketkai B.N., Haydek J.P., Agrawal M., Scott F.I., Ananthakrishnan A.N. (2024). AGA Living Clinical Practice Guideline on Pharmacological Management of Moderate-to-Severe Ulcerative Colitis. Gastroenterology.

[7] Nagaraj T., Shinn J., De Felice K. (2024). A practical guide to selecting and using new ulcerative colitis therapies. Curr. Opin. Gastroenterol..

[8] Singh S., Allegretti J.R., Siddique S.M., Terdiman J.P. (2020). AGA Technical Review on the Management of Moderate to Severe Ulcerative Colitis. Gastroenterology.

[9] Lelièvre O., Benoist S., Brouquet A. (2024). Indications, modalities, and outcomes of surgery for ulcerative colitis in 2024. J. Visc. Surg..

[10] Buie M.J., Quan J., Windsor J.W., Coward S., Hansen T.M., King J.A., Kotze P.G., Gearry R.B., Ng S.C., Mak J.W.Y. (2023). Global Hospitalization Trends for Crohn’s Disease and Ulcerative Colitis in the 21st Century: A Systematic Review With Temporal Analyses. Clin. Gastroenterol. Hepatol..

[11] Danese S., Vermeire S., Zhou W., Pangan A.L., Siffledeen J., Greenbloom S., Hébuterne X., D’Haens G., Nakase H., Panés J. (2022). Upadacitinib as induction and maintenance therapy for moderately to severely active ulcerative colitis: Results from three phase 3, multicentre, double-blind, randomised trials. Lancet.

[12] Raine T., Bonovas S., Burisch J., Kucharzik T., Adamina M., Annese V., Bachmann O., Bettenworth D., Chaparro M., Czuber-Dochan W. (2022). ECCO Guidelines on Therapeutics in Ulcerative Colitis: Medical Treatment. J. Crohn’s Colitis.

[13] Burr N.E., Gracie D.J., Black C.J., Ford A.C. (2021). Efficacy of biological therapies and small molecules in moderate to severe ulcerative colitis: Systematic review and network meta-analysis. Gut.

[14] Ahuja D., Murad M.H., Ma C., Jairath V., Singh S. (2023). Comparative Speed of Early Symptomatic Remission With Advanced Therapies for Moderate-to-Severe Ulcerative Colitis: A Systematic Review and Network Meta-Analysis. Am. J. Gastroenterol..

[15] Shehab M., Hassan A., Alrashed F., Abbas A., Ma C., Narula N., Jairath V., Singh S., Bessissow T. (2025). Comparative Efficacy of Biologics and Small Molecule Therapies in Improving Patient-Reported Outcomes in Ulcerative Colitis: Systematic Review and Network Meta-Analysis. Inflamm. Bowel Dis..

[16] Friedberg S., Choi D., Hunold T., Choi N.K., Garcia N.M., Picker E.A., Cohen N.A., Cohen R.D., Dalal S.R., Pekow J. (2023). Upadacitinib Is Effective and Safe in Both Ulcerative Colitis and Crohn’s Disease: Prospective Real-World Experience. Clin. Gastroenterol. Hepatol..

[17] Kochhar G.S., Khataniar H., Jairath V., Farraye F.A., Desai A. (2024). Comparative Effectiveness of Upadacitinib and Tofacitinib in Ulcerative Colitis: A US Propensity-Matched Cohort Study. Am. J. Gastroenterol..

[18] Nogami A., Asonuma K., Okabayashi S., Ikenouchi M., Matsuda T., Shinzaki S., Fukata M., Kobayashi T. (2024). Real-World Comparative Effectiveness and Safety of Filgotinib and Upadacitinib for Ulcerative Colitis: A Multicentre Cohort Study. United Eur. Gastroenterol. J..

[19] Dalal R.S., Kallumkal G., Cabral H.J., Barnes E.L., Allegretti J.R. (2024). One-Year Comparative Effectiveness of Upadacitinib vs Tofacitinib for Ulcerative Colitis: A Multicenter Cohort Study. Am. J. Gastroenterol..

[20] MDV Database Overview. https://en.mdv.co.jp/ebm/about-mdv-database/mdv-database-overview/.

[21] Wong E.C.L., Jairath V., Dulai P.S., Marshall J.K., Ma C., Reinisch W., Narula N. (2026). Infliximab and Upadacitinib Demonstrate Superior Early Onset of Efficacy in Biologic-Naïve Ulcerative Colitis with Severe Endoscopic Disease. Am. J. Gastroenterol..

[22] Loftus E.V., Colombel J.F., Takeuchi K., Gao X., Panaccione R., Danese S., Dubinsky M., Schreiber S., Ilo D., Finney-Hayward T. (2023). Upadacitinib Therapy Reduces Ulcerative Colitis Symptoms as Early as Day 1 of Induction Treatment. Clin. Gastroenterol. Hepatol..

[23] Prieto-Merino D., Mulick A., Armstrong C., Hoult H., Fawcett S., Eliasson L., Clifford S. (2021). Estimating proportion of days covered (PDC) using real-world online medicine suppliers’ datasets. J. Pharm. Policy Pract..

[24] Gilmore R., Tan W.L., Fernandes R., An Y.K., Begun J. (2023). Upadacitinib Salvage Therapy for Infliximab-Experienced Patients with Acute Severe Ulcerative Colitis. J. Crohn’s Colitis.

[25] Panés J., Dubinsky M.C., Ishiguro Y., Shukla N., Dubcenco E., Remple V., Sharma D., Panaccione R. (2025). Achievement of long-term treatment goals in upadacitinib-treated patients with moderately to severely active ulcerative colitis: A post hoc analysis of phase 3 trial data. J. Crohn’s Colitis.

[26] Panaccione R., Vermeire S., Danese S., Higgins P.D.R., Lichtenstein G.R., Nakase H., Glover S., Colombel J.F., Eccleston J., Kujawski M. (2025). Long-term efficacy and safety of upadacitinib in patients with moderately to severely active ulcerative colitis: An interim analysis of the phase 3 U-ACTIVATE long-term extension study. Lancet Gastroenterol. Hepatol..

[27] Sandborn W.J., Ghosh S., Panes J., Schreiber S., D’Haens G., Tanida S., Siffledeen J., Enejosa J., Zhou W., Othman A.A. (2020). Efficacy of Upadacitinib in a Randomized Trial of Patients With Active Ulcerative Colitis. Gastroenterology.

[28] LeBlanc K., Mosli M.H., Parker C.E., MacDonald J.K. (2015). The impact of biological interventions for ulcerative colitis on health-related quality of life. Cochrane Database Syst. Rev..

[29] Loftus E.V., Panés J., Lacerda A.P., Peyrin-Biroulet L., D’Haens G., Panaccione R., Reinisch W., Louis E., Chen M., Nakase H. (2023). Upadacitinib Induction and Maintenance Therapy for Crohn’s Disease. N. Engl. J. Med..

[30] Genovese M.C., Fleischmann R., Combe B., Hall S., Rubbert-Roth A., Zhang Y., Zhou Y., Mohamed M.F., Meerwein S., Pangan A.L. (2018). Safety and efficacy of upadacitinib in patients with active rheumatoid arthritis refractory to biologic disease-modifying anti-rheumatic drugs (SELECT-BEYOND): A double-blind, randomised controlled phase 3 trial. Lancet.

[31] McInnes I.B., Anderson J.K., Magrey M., Merola J.F., Liu Y., Kishimoto M., Jeka S., Pacheco-Tena C., Wang X., Chen L. (2021). Trial of Upadacitinib and Adalimumab for Psoriatic Arthritis. N. Engl. J. Med..

[32] van der Heijde D., Song I.H., Pangan A.L., Deodhar A., van den Bosch F., Maksymowych W.P., Kim T.H., Kishimoto M., Everding A., Sui Y. (2019). Efficacy and safety of upadacitinib in patients with active ankylosing spondylitis (SELECT-AXIS 1): A multicentre, randomised, double-blind, placebo-controlled, phase 2/3 trial. Lancet.

[33] Silverberg J.I., Bunick C.G., Hong H.C., Mendes-Bastos P., Stein Gold L., Costanzo A., Ibrahim N., Sancho C., Wu X., Han Y. (2024). Efficacy and safety of upadacitinib versus dupilumab in adults and adolescents with moderate-to-severe atopic dermatitis: Week 16 results of an open-label randomized efficacy assessor-blinded head-to-head phase IIIb/IV study (Level Up). Br. J. Dermatol..

[34] Yerushalmy-Feler A., Spencer E.A., Dolinger M.T., Suskind D.L., Mitrova K., Hradsky O., Conrad M.A., Kelsen J.R., Uhlig H.H., Tzivinikos C. (2025). Upadacitinib for Induction of Remission in Pediatric Ulcerative Colitis: An International Multicenter Study. J. Crohn’s Colitis.

[35] Moran G.W., Gordon M., Sinopolou V., Radford S.J., Darie A.M., Vuyyuru S.K., Alrubaiy L., Arebi N., Blackwell J., Butler T.D. (2025). British Society of Gastroenterology guidelines on inflammatory bowel disease in adults: 2025. Gut.

